# Distinct telomere differences within a reproductively bimodal common lizard population

**DOI:** 10.1111/1365-2435.13408

**Published:** 2019-07-30

**Authors:** Darryl McLennan, Hans Recknagel, Kathryn R. Elmer, Pat Monaghan

**Affiliations:** ^1^ Institute of Biodiversity, Animal Health and Comparative Medicine University of Glasgow Glasgow UK; ^2^ Department of Fish Ecology and Evolution EAWAG Kastanienbaum Switzerland

**Keywords:** life‐history variation, oviparity, physiological state, squamate, viviparity

## Abstract

1. Different strategies of reproductive mode, either oviparity (egg‐laying) or viviparity (live‐bearing), will be associated with a range of other life‐history differences that are expected to affect patterns of ageing and longevity. It is usually difficult to compare the effects of alternative reproductive modes because of evolutionary and ecological divergence. However, the very rare exemplars of reproductive bimodality, in which different modes exist within a single species, offer an opportunity for robust and controlled comparisons.
2. One trait of interest that could be associated with life history, ageing and longevity is the length of the telomeres, which form protective caps at the chromosome ends and are generally considered a good indicator of cellular health. The shortening of these telomeres has been linked to stressful conditions; therefore, it is possible that differing reproductive costs will influence patterns of telomere loss. This is important because a number of studies have linked a shorter telomere length to reduced survival.
3. Here, we have studied maternal and offspring telomere dynamics in the common lizard (*Zootoca vivipara*). Our study has focused on a population where oviparous and viviparous individuals co‐occur in the same habitat and occasionally interbreed to form admixed individuals.
4. While viviparity confers many advantages for offspring, it might also incur substantial costs for the mother, for example require more energy. Therefore, we predicted that viviparous mothers would have relatively shorter telomeres than oviparous mothers, with admixed mothers having intermediate telomere lengths. There is thought to be a heritable component to telomere length; therefore, we also hypothesized that offspring would follow the same pattern as the mothers.
5. Contrary to our predictions, the viviparous mothers and offspring had the longest telomeres, and the oviparous mothers and offspring had the shortest telomeres. The differing telomere lengths may have evolved as an effect of the life‐history divergence between the reproductive modes, for example due to the increased growth rate that viviparous individuals may undergo to reach a similar size at reproduction.

A free http://onlinelibrary.wiley.com/doi/10.1111/1365-2435.13408/suppinfo can be found within the Supporting Information of this article.

## Graphical abstract

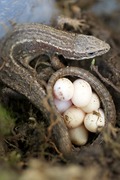

## 1 INTRODUCTION

Differing reproductive strategies can co‐occur within a species, due to both environmental and genetic differences among individuals, as well as the interaction between the two (Taborsky & Brockmann, 2010). This phenotypic variation can exist on a continuous spectrum or fall into discontinuous life‐history modes (Partridge, MacManes, Knapp, & Neff, 2016; Tsubaki, Hooper, & Siva‐Jothy, 1997), and strategies can be plastic or fixed within an individual's lifetime (Bailey, Gray, & Zuk, 2010; Baum, Laughton, Armstrong, & Metcalfe, 2004; Bronikowski & Arnold, 1999; Meunier et al., 2012; Zamudio & Chan, 2008).

A striking example of fixed, discontinuous reproductive modes is when a species is bimodal for both oviparity (i.e. egg‐laying) and viviparity (i.e. giving birth to live young). Viviparity is evolutionarily derived from oviparity and confers many benefits to offspring, particularly a more stable and protected developmental environment in the mother's reproductive tract (Shine, 1995). However, viviparity can also restrict a mother's reproductive output and may reduce the number of clutches produced within a given year (Blackburn, 1999; Recknagel & Elmer, 2019; Sites, Reeder, & Wiens, 2011; Wourms & Lombardi, 1992). Most vertebrate species exhibit only one of these reproductive modes; however, in a small number of species, both reproductive modes occur (Murphy & Thompson, 2011). The differing maternal costs associated with each reproductive mode have been little studied, in part because so few species exhibit both modes. However, it has been suggested that viviparity may incur substantial costs for the mother. Firstly, the prolonged gestational period and presumed larger clutch mass in viviparous individuals (Horváthová et al., 2013; Qualls & Shine, 1998; Roitberg et al., 2013) could carry a greater metabolic cost for the mother. Secondly, viviparity has been linked to the evolution of larger body sizes to counteract the space constraint *in utero* (Qualls & Shine, 1995) and there can be costs associated with rapid growth and/or a larger body size (Metcalfe & Monaghan, 2003).

It is likely that such a divergence in reproductive mode will also be associated with other life‐history differences, which could in turn affect patterns of ageing and longevity. One trait that could be of interest in this context is telomere length. Telomeres cap the ends of eukaryotic chromosomes and play an important role in chromosome protection (Blackburn, 1991; Monaghan, 2010). These telomere caps shorten with each round of cell division because of the ‘end replication problem’ (Levy, Allsopp, Futcher, Greider, & Harley, 1992). Certain species are capable of telomere elongation, mostly via the expression of the enzyme telomerase (Gomes, Shay, & Wright, 2010; Tian et al., 2018). However, in the absence of elongation mechanisms, telomeres may shorten to such an extent that the central coding region of the chromosome becomes vulnerable. As such, a relatively short telomere length is considered to be an indicator of poor cellular and biological state, and a number of studies have linked a shorter telomere length and/or a faster rate of telomere attrition to reduced survival and/or longevity (Boonekamp, Mulder, Salomons, Dijkstra, & Verhulst, 2014; Debes, Visse, Panda, Ilmonen, & Vasemagi, 2016; Dupoué et al., 2017; Salmón, Nilsson, Nord, Bensch, & Isaksson, 2016; Wilbourn et al., 2018).

In addition to cell division, the accelerated erosion of telomeres has been linked to environmental stressors, potentially via oxidative stress pathways (Monaghan & Ozanne, 2018; Reichert & Stier, 2017, but see also Boonekamp, Bauch, Mulder, & Verhulst, 2017). Recent studies have linked telomere dynamics to various stressful conditions, both in vitro and in vivo (Barnes, Fouquerel, & Opresko, 2018; Cram, Monaghan, Gillespie, & Clutton‐Brock, 2017; Debes et al., 2016; Monaghan, 2014; Olsson et al., 2018). Moreover, a recent study on Australian painted dragons (*Ctenophorus pictus*) found that telomere dynamics differed between individuals with different reproductive and life‐history tactics (Rollings et al., 2017). It is therefore possible that differing reproductive costs associated with oviparity or viviparity will also influence patterns of telomere loss.

In addition to affecting maternal telomere length, differences between oviparous and viviparous life‐history strategies could also affect the telomere length of the offspring that arise from these reproductive modes. A number of studies have reported a heritable component to telomere length (Bouwhuis, Verhulst, Bauch, & Vedder, 2018; Dugdale & Richardson, 2018). Moreover, offspring telomere length could also be subjected to maternal effects, for example via differences in embryonic provisioning and/or embryonic environment (McLennan et al., 2018a; Noguera, Metcalfe, Reichert, & Monaghan, 2016), as well as potential maternal effects on oocyte telomere length (Keefe, Kumar, & Kalmbach, 2015). Further, since oviparous offspring interact with the environment at an earlier life stage, it is possible that the telomeres of these offspring could then be differentially affected by environmental factors, for example via temperature and growth effects on early life development (McLennan et al., 2018b; Monaghan & Ozanne, 2018; Vedder, Verhulst, Zuidersma, & Bouwhuis, 2018).

The common lizard (*Zootoca vivipara*) is one of only a few extant vertebrate species in which both viviparity and oviparity occur (Surget‐Groba et al., 2006). In this study, we examine maternal and offspring telomere dynamics in a wild common lizard population in which different evolutionary lineages with either an oviparous or viviparous reproductive mode coexist within the same habitat. Reproductive mode in this species is known to be genetically determined and fixed between lineages (Arrayago, Bea, & Heulin, 1996; Recknagel & Elmer, 2019; Recknagel, Kamenos, & Elmer, 2018). The oviparous mothers lay calcified eggs ~33 days after copulation (i.e. oviposition) with a mean thickness of 40 µm, which are then incubated by the mother for ~28 days (Arrayago et al., 1996; Heulin, 1990; Lindtke, Mayer, & Böhme, 2010). Offspring from viviparous mothers are retained in utero for the duration of embryonic development (~57 days between copulation and birth; Arrayago et al., 1996) and are fully developed at birth (i.e. parturition). Each viviparous offspring is born individually surrounded by a thin membrane, from which they then ‘hatch out’ of immediately or within a few days after parturition (Recknagel & Elmer, 2019). Because this population occurs in a unique contact zone between the two reproductive modes, admixture is also possible (Lindtke et al., 2010; Recknagel, 2018). Admixed offspring are created when interbreeding occurs between the two reproductive modes. These offspring may be first‐generation hybrids or result from the backcrossing of hybrids with oviparous or viviparous individuals. Offspring from admixed individuals are laid in thinner and less calcified eggs, but at a later developmental stage, compared with oviparous offspring. However, embryo mortality is also much higher in the admixed offspring, with estimates of around 40% (Lindtke et al., 2010).

Here, we have measured telomere length in viviparous, oviparous and admixed common lizards, of both mothers and their offspring. We predicted that viviparous mothers (with the presumption that they incur a higher reproductive burden) would have relatively shorter telomeres than oviparous mothers, while the telomere lengths of admixed mothers would be intermediate between their oviparous and viviparous conspecifics. Lastly, since there is known to be a heritable component to telomere length, we predicted that the patterns of telomere length in the offspring would follow those of the mothers.

## 2 MATERIALS AND METHODS

### 2.1 Field study

The field aspect of this study was conducted in the Gailtal valley of the Carinthian Alps, Austria (N 46.60°, E 13.14°), and under permit number HE3‐NS‐959/2013 (012/2016). This is currently the only known location where both viviparous and oviparous common lizards co‐occur in high densities and occasionally interbreed (Cornetti et al., 2015; Lindtke et al., 2010; Recknagel, 2018). The study site covered an area of approximately 0.3 km^2^ and an altitudinal range of 1,380–1,580 m. Wild female lizards were caught between May and July 2016; females can be distinguished from males by the absence of a hemipenal bulge at the base of the tail. Whether or not a female had recently mated was identified by the presence of a biting mark on the female's belly or flank (Lindtke et al., 2010). Immediately after capture, all lizards were weighed (to the nearest 0.001 g) and snout–vent length (SVL) and tail length (TL) were measured (to the nearest 0.01 mm). Tail autotomy (self‐amputation of the tail as a defence from predators and conspecifics) is a common occurrence in this species. Tail autotomy has previously been linked to telomere dynamics (Olsson, Pauliny, Wapstra, & Blomqvist, 2010); therefore, for this study, we decided to include only females that had a non‐autotomized tail.

Females that had recently mated (and were therefore likely to be pregnant) were moved to nearby holding facilities so that their reproductive mode could be assessed. All females were individually housed in plastic terraria (56 × 39 × 28 cm) that were covered by netting on the top and on one side, to allow sufficient airflow. The terraria were housed within tents, so that each lizard was exposed to natural temperature variation. Each terrarium contained sand substrate, suitable shelter (e.g. pieces of dried wood), moisturized moss and a bowl of water. Lizards were fed ad libitum with mealworms (*Tenebrio molitor*) and crickets (*Gryllus assimilis*).

Each female was checked daily for the presence of offspring. On the same day that a female had undergone oviposition (to shelled eggs) or parturition (to live young), the female was weighed (to the nearest 0.001 g) and the number of offspring within a clutch (hereafter clutch size, CZ), relative clutch mass (RCM: clutch mass, including eggshell, amniotic fluids and yolk, divided by female mass after oviposition/parturition) and relative offspring mass (ROM: summed mass of the offspring after hatching, excluding eggshell, amniotic fluids and yolk, divided by female mass after oviposition/parturition) was measured. After oviposition/parturition, a tail clip was taken from each female for subsequent sequencing and telomere analysis. While absolute telomere lengths may differ among tissues, studies have found strong correlations in telomere length between different tissues in birds (Reichert, Criscuolo, Verinaud, Zahn, & Massemin, 2013) and lizards (Rollings et al., 2019). Moreover, a study on brown trout *Salmo trutta* by Debes et al. (2016) found trends in telomere dynamics to be similar among tissues, including highly regenerative tissues such as fin. Therefore, we were confident in using the tail clips for a non‐invasive measurement of telomere length. Shortly after sampling, the females were returned and released back to the same location at which they had been caught. Unhatched offspring were incubated at 24°C in an Exo Terra Incubator. On the day of birth or hatching, each individual was weighed (to the nearest 0.001 g), SVL was measured (to the nearest 0.01 mm), and a tail clip was taken for subsequent telomere analysis. The offspring were then returned and released back to the same location at which the mothers had been caught. In total, we had complete tail samples for 69 mothers (23 oviparous, 33 viviparous and 13 admixed individuals). Offspring that did not hatch were excluded. In total, we had offspring tail clip samples from 57 of the mothers (20 oviparous, 29 viviparous and 8 admixed individuals).

### 2.2 DNA extraction

The tail clip samples were transferred to the University of Glasgow, UK. DNA was extracted from all samples using the Macherey‐Nagel DNA NucleoSpin Tissue Extraction Kit, following the manufacturer's protocol. For each of the mothers, a cross‐sectional sample of the tail (approximately 3 mm wide) was incubated in 180 µl lysis buffer + 20 µl of proteinase K solution (20 mg/ml) at 56°C overnight. Samples were then centrifuged to separate bone fragments from the tissue lysate, with the lysate being used in the subsequent DNA extraction. For the offspring, all siblings within a clutch were pooled (min 1 offspring, max 10 offspring) so that there was one averaged offspring DNA sample per mother. Again, a cross‐sectional sample of tail (approximately 1 mm wide) was taken from each of the siblings and the pooled tissue was then processed in the same way as for the mothers. DNA concentration and purity was measured spectrophotometrically using a NanoDrop 8000, which confirmed that all samples met the recommended A260/280 ratio and had a DNA concentration >20 ng/µl.

### 2.3 Identifying maternal reproductive mode by ddRADSeq

Oviparous and viviparous females are easily distinguishable by phenotype: oviparous offspring are laid in calcified shells and then require ~28 days of incubation prior to hatching, while viviparous offspring are born fully developed, but in a thin membrane that they then ‘hatch out’ of within a few hours to days after birth (Arrayago et al., 1996; Lindtke et al., 2010). However, because our study population occurs in a unique contact zone, there were also females that laid admixed offspring that exhibited intermediate phenotypes, such as partially calcified shells and fewer days of incubation compared to oviparous clutches. Therefore, we used a double‐digest restriction site‐associated sequencing (ddRADSeq) approach to genetically distinguish between the reproductive modes and admixed individuals. To do so, we followed the ddRADSeq library preparation protocol of Recknagel et al. (2018); see Appendix S1 for details.

Females were assigned a membership value (*Q*) to establish from which lineage of which reproductive mode they derived (Recknagel et al., 2018). Females that had a strong signature of oviparous genomic background (*Q* value ≤0.01) were assigned as oviparous, while females with a strong viviparous genomic background (*Q* value ≥0.99) were assigned as viviparous. Females that had a genomic background of admixture (*Q* value >0.01 and <0.99) were assigned as admixed individuals. We assigned a reproductive mode to 67 out of 69 of the females based on their genotypes. Two of the lizards were not included in the ddRADSeq library; however, their number of incubation days was clearly in the viviparous and oviparous ranges (2 and 35, respectively); therefore, we were confident in assigning their reproductive mode based on phenotype alone.

### 2.4 Telomere analysis

Common lizard telomere length has been previously measured using the TeloTAGGG Telomere Length Assay (Dupoué et al., 2017), confirming that common lizard telomeres are also made up of the repetitive sequence TTAGGG. For this study, telomere length was measured in all samples using the quantitative PCR method described by Cawthon (2002). The universal Tel1b and Tel2b primers designed by Cawthon (2002) and modified by Epel et al. (2004) were used for amplification of the telomere repeats. The recombination activating gene 1 (RAG‐1) was chosen as the single‐copy gene, and the *Z. vivipara* RAG‐1 sequence (GenBank accession number: KY762205.1) was used to design primers. The following forward and reverse RAG‐1 primers successfully amplified a single amplicon, as determined by melt curve analysis, and were subsequently used in the analysis:

LizRAG1‐F 5′‐GCC AAC TGC AAC AAG ATA CAC‐3′ and LizRAG1‐R 5′‐GAT ATG CTC ACA GAC CTG ACA A‐3′.

A full outline of the qPCR protocol is provided in the Appendix S1. The samples (69 mothers and 57 offspring) were randomly distributed across six sets of PCR plates. qPCR data were analysed using the qbase software for Windows (Hellemans, Mortier, Paepe, Speleman, & Vandesompele, 2007), as described in McLennan et al. (2016). For each sample, the qbase software produced a calibrated normalized relative quantity (CNRQ). This is similar to the T/S ratio described by Cawthon (2002) but with greater control of the qPCR efficiency and inter‐plate variation (see Appendix S1 for further details). Three points from the standard curve (5, 10 and 20 ng/well) were used as inter‐run calibrators during qBASE analysis, to help correct for inter‐run variation. The remaining three points of the standard curve (40, 2.5 and 1.25 ng/well) were used to calculate an inter‐assay coefficient of variability of the CNRQs (which was 8.22). The average intra‐plate variation of the Ct values was 0.94 for the telomere assay and 0.33 for the RAG‐1 assay, respectively. The average inter‐plate variation of the Ct values was 1.44 for the telomere assay and 0.34 for the RAG‐1 assay, respectively. The efficiencies of the telomere and RAG‐1 assays ranged from 95.6% to 108.5% and 91.7% to 97.9%, respectively, and were therefore within the acceptable range (85%–115%). The average quantification cycle (Ct) for the telomere and RAG‐1 assays was 12.04 and 22.87, respectively. We found no significant difference in the RAG‐1 Ct values among the three reproductive modes (oviparous, viviparous and admixed) (GLM *F*_2,66_ = 1.08, *p* = .35), but a highly significant difference among the telomere Ct values (GLM *F*_2,66_ = 19.08, *p* < .001).

### 2.5 Statistical analysis

We used a Pearson correlation coefficient matrix to assess potential collinearity between all covariates (with a cut‐off coefficient of 0.8). Mass and length were highly collinear for both mothers (Pearson *r* = .91, *p* < .001) and offspring (Pearson *r* = .91, *p* < .001); therefore, only mass was used for subsequent analyses. All variables used in the analyses are shown in Table 1.

**Table 1:** Summary of all the variables used in statistical analyses

Variable name	Variable description
Life stage	Life stage at which a sample was taken (i.e. whether from mother or offspring). Factor
Reproductive mode	Reproductive phenotype of each mother, based on the ddRADSeq analysis. Offspring were assigned the same mode as their mother. Factor
Maternal RTL	Relative telomere length of each mother, measured at the individual level. Covariate
Mean offspring RTL	Relative offspring telomere length, measured as an average for each mother across all offspring. Covariate
Maternal mass	Somatic mass of each mother after oviposition or parturition (to the nearest 0.001 g). Covariate
Offspring mass	Somatic mass of offspring at the time of hatching, measured as an average for each mother (to the nearest 0.001 g). Covariate
RCM	Relative clutch mass: clutch mass divided by female mass after oviposition or parturition. Covariate
ROM	Relative offspring mass: sum of the offspring mass after hatching divided by female mass after oviposition/parturition. Covariate
Clutch size	Number of offspring born within a given clutch. Covariate

Note

See Section 22 for an outline of each model.

All statistical analyses were conducted using r version 3.5.0 software. In total, we ran eight statistical models. Firstly, we ran general linear models (GLMs) to assess whether maternal mass (model 1), offspring mass (model 2), clutch size (model 3) and relative clutch mass RCM (model 4) and relative offspring mass ROM (model 5) varied between the female reproductive mode. Secondly, we conducted a linear mixed model (LME) using the lme4 and lmerTest functions (Bates, Maechler, Bolker, & Walker, 2015; Kuznetsova, Brockhoff, & Christensen, 2014) to assess whether RTL (relative telomere length) differed between life stage and reproductive mode, which included family ID as a random factor to control for non‐independence between mother and offspring (model 6). Estimates of marginal (fixed effects) and conditional (fixed effects + random effects) *R* ^2^ values of the mixed model were calculated using the MuMin package. Finally, we looked at factors affecting variation in RTL at each life stage by conducting two separate GLMs. Firstly, maternal RTL was assessed in relation to reproductive mode, maternal mass, clutch size and RCM (model 7). This model was then simplified by backwards elimination, starting with the most insignificant term and continuing with insignificant main effects until the model contained only significant terms; providing that this resulted in a reduction of the AIC score. Secondly, mean offspring RTL was assessed in relation to maternal RTL, reproductive mode, offspring mass, clutch size, RCM and the interaction maternal RTL with reproductive mode to assess whether the relationship between maternal RTL and mean offspring RTL differed between the reproductive modes (model 8). As before, model 8 was then simplified by backwards elimination, starting with insignificant interactions and continuing with insignificant main effects until the model contained only significant terms. Variables were only removed from the model if this resulted in a reduction of the Akaike information criterion (AIC) score.

## 3 RESULTS

### 3.1 Life‐history variation

**Figure 1:**
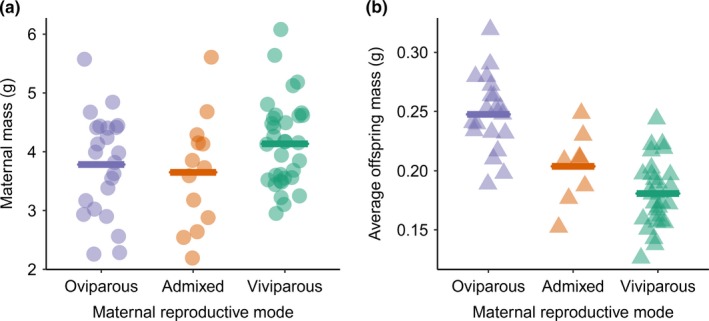
The relationship between maternal reproductive mode and the somatic mass of (a) mothers and (b) offspring. Data plotted as individuals + mean. Somatic mass did not differ significantly between reproductive modes at the maternal stage; however, there was a significant difference at the offspring stage, measured on the day of birth or hatching (*p* < .05; see Table 2)

**Figure 2:**
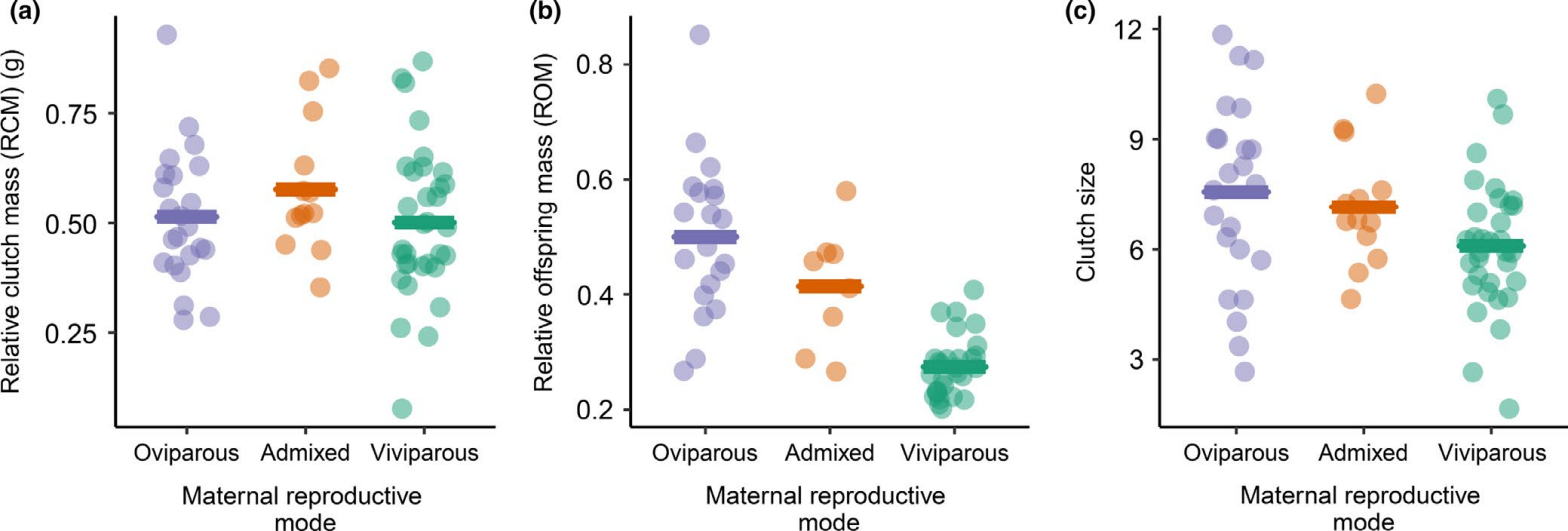
The relationship between maternal reproductive mode and (a) relative clutch mass (clutch mass, including eggshell, amniotic fluids and yolk, divided by female mass after oviposition/parturition), (b) relative offspring mass (summed mass of the offspring after hatching, excluding eggshell, amniotic fluids and yolk, divided by female mass after oviposition/parturition) and (c) the number of offspring within a clutch. Data plotted as individuals + mean. RCM did not differ between the reproductive modes; however, there was a significant reproductive mode effect on ROM and clutch size (*p* < .05; see Table 2)

We did not find a significant difference in somatic mass among the reproductive modes at the maternal stage, measured after oviposition/parturition (Table 2.1 and Figure 1a); however, there was a significant difference at the offspring stage, measured on the day of birth or hatching, with oviparous offspring being the heaviest, viviparous offspring being the lightest and admixed offspring being the intermediate (GLM *F*_2,54_ = 30.85, *p* < .001; Table 2.2 and Figure 1b). The number of offspring within a clutch also differed significantly among the reproductive modes, with viviparous mothers producing the smallest clutches and oviparous mothers producing the largest clutches (GLM *F*_2,66_ = 3.99, *p* = .023; Table 2.3 and Figure 2c). RCM (relative clutch mass, including eggshell, amniotic fluids, etc.) did not significantly differ among the modes, suggesting that there was similar resource investment among the mothers (Table 2.4). However, we did find a significant difference in ROM (relative offspring mass, excluding eggshell, amniotic fluids, etc.) among the reproductive modes, with ROM being highest in the oviparous families and lowest in the viviparous families (GLM *F*_2,54_ = 33.15, *p* < .001; Table 2.5 and Figure 2b). This suggests that while there was a similar investment of maternal resources among the reproductive modes (in terms of embryo, eggshell, yolk and amniotic fluid production), the oviparous females had a higher net gain per clutch, supported by the larger clutch size and offspring mass.

**Table 2:** Summary of the final GLMs corresponding to models 1, 2, 3, 4, 5, 7 and 8

#		Explanatory variable	Estimate	*SE*	*t*	*p*
1	Maternal mass	—	—	—	—	—
2	Offspring mass	Intercept	0.204	0.010	19.67	<.001
		Repro. mode—oviparous	0.044	0.012	3.57	<.001
		Repro. mode—viviparous	−0.023	0.012	−1.97	.054
3	Clutch size	Intercept	7.154	0.553	12.94	<.001
		Repro. mode—oviparous	0.411	0.691	0.60	.554
		Repro. mode—viviparous	−1.063	0.653	−1.63	.108
4	RCM	—	—	—	—	—
5	ROM	Intercept	0.414	0.034	12.11	<.001
		Repro. mode—oviparous	0.086	0.040	2.13	.037
		Repro. mode—viviparous	−0.140	0.039	−3.62	<.001
7	Maternal RTL	Intercept	0.043	0.033	1.28	.204
		Repro. mode—oviparous	−0.166	0.041	−3.98	<.001
		Repro. mode—viviparous	0.118	0.039	3.01	<.001
8	Mean offspring RTL	Intercept	−0.077	0.030	−2.52	.014
		Repro. mode—oviparous	−0.135	0.036	−3.73	<.001
		Repro. mode—viviparous	0.146	0.034	4.24	<.001

Note

See Section 22 for full definitions of the main effects and interactions initially included in each model. See Section 33 for analysis of variance test statistics.

### 3.2 Telomere length variation

**Figure 3:**
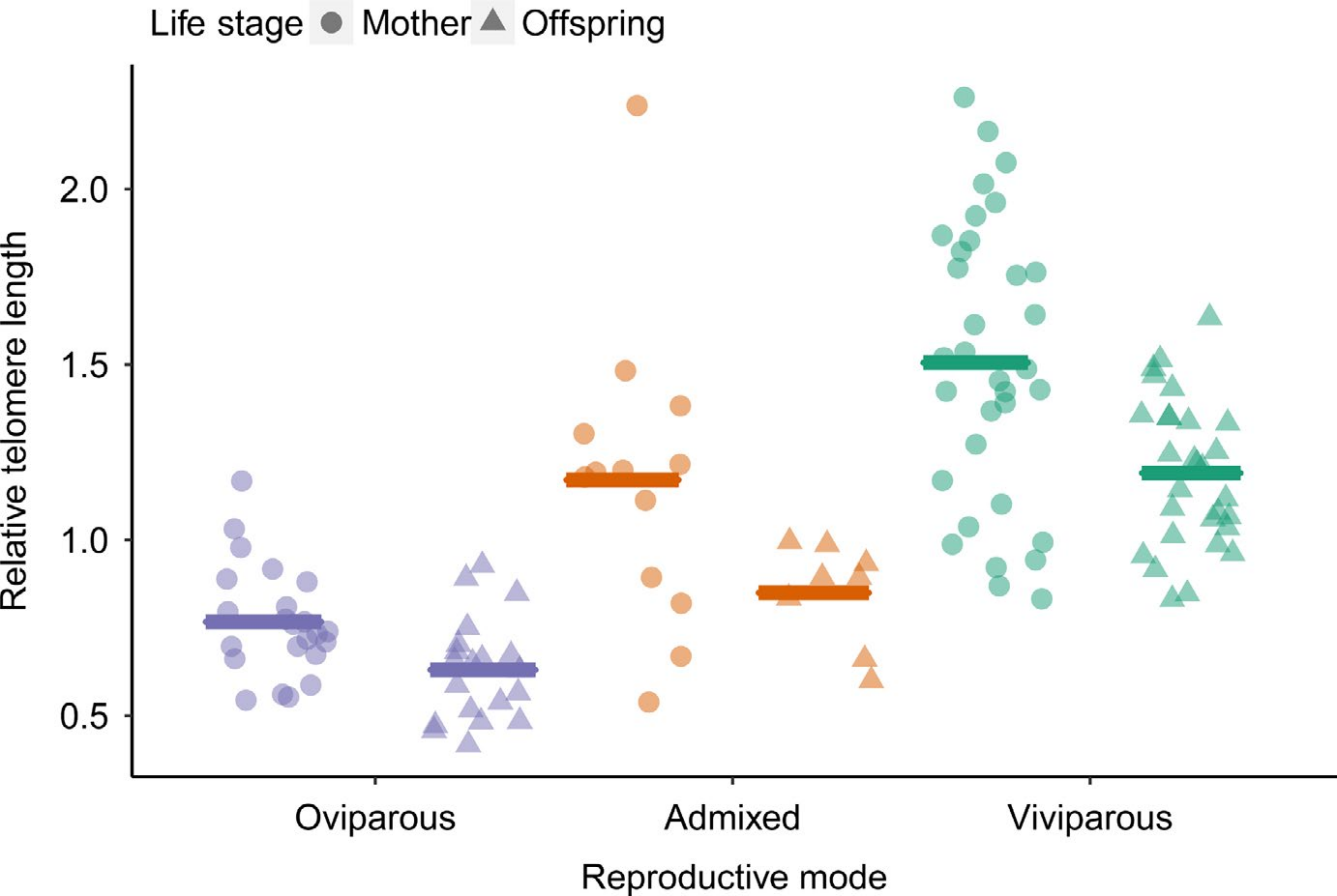
The relationship between reproductive mode and relative telomere length (RTL). Circles correspond to mothers, while triangles correspond to the offspring. Data plotted as individuals + mean

There was a significant difference in RTL between mothers and offspring. Mothers had relatively longer telomeres than their offspring within each of the three reproductive modes (LMM *F*_1,55.03_ = 32.14, *p* < .001; Table 3 and Figure 3). Telomere length also differed significantly between the reproductive modes. The viviparous mode had the longest telomeres, the oviparous mode had the shortest telomeres, and the admixed individuals had relatively intermediate telomeres (LMM *F*_2,59.13_ = 72.55, *p* < .001; Table 3 and Figure 3). This was true for both mothers (GLM *F*_2,66_ = 37.98, *p* < .001; Table 2.7 and Figure 3) and offspring (GLM *F*_2,54_ = 63.12, *p* < .001; Table 2.8 and Figure 3). When focusing only on the mothers, maternal mass, clutch size and RCM were not significantly associated with maternal RTL. For the offspring, maternal RTL, offspring mass, clutch size, RCM and the interaction maternal RTL X reproductive mode were not significantly associated with the relative mean telomere length.

**Table 3:** Summary of the linear mixed‐effect model, corresponding to model 6

#		Explanatory variable	Estimate	*SE*	*df*	*t*	*p*
6	RTL	Intercept	0.035	0.025	74.78	1.35	.18
		Repro. mode—oviparous	−0.155	0.031	62.04	−5.00	<.001
		Repro. mode—viviparous	0.127	0.029	62.80	4.32	<.001
		Life stage—offspring	−0.094	0.017	55.03	−4.67	<.001

Note

Family ID was included as a random factor to control for non‐independence between mother and offspring. The family level variance was 0.0029 (*SD* 0.0542), and the residual variance was 0.0083 (*SD* 0.091). See Section 33 for analysis of variance test statistics. Estimates of marginal (fixed effects) and conditional (fixed effects + random effects) *R* ^2^ values were 0.62 and 0.72, respectively.

## 4 DISCUSSION

This study has shown that different reproductive modes of the common lizard significantly differ in telomere length. The viviparous mode had the longest telomeres (for both mothers and offspring), the oviparous mode had the shortest telomeres (for both mothers and offspring), while the admixed individuals had relatively intermediate telomeres (again, for both mothers and offspring). Since the significant difference in telomere length was already evident at the offspring stage, it suggests that the reproductive modes have evolved differing telomere dynamics over time. However, it is currently unclear whether and/or how these differences in telomere length have co‐evolved with other life‐history traits. We also found that mothers had relatively longer telomeres than their offspring among the reproductive ecotypes. However, we currently do not know whether this is due to physiological processes (e.g. telomere elongation mechanisms during development; Gomes et al., 2010) or due to the selective disappearance of individuals born with relatively smaller telomeres (e.g. Salmón, Nilsson, Watson, Bensch, & Isaksson, 2017).

We initially hypothesized that the viviparous mothers would have a higher reproductive investment (Lindtke et al., 2010) because of the prolonged pregnancy and presumed larger clutch mass in viviparous species (Horváthová et al., 2013; Qualls & Shine, 1998; Roitberg et al., 2013). However, the relative clutch mass (as a proxy for reproductive investment) was similar between the reproductive modes. Another potential cost to viviparity is that viviparous females may be less able to hunt and acquire resources during pregnancy, which could result in a net loss of energy. However, this was not possible to quantify in this study, since the pregnant females were fed ad libitum. It has also been suggested that viviparous species have longer life spans, compared to oviparous species, and are therefore able to produce more clutches over the years (Meiri, Brown, & Sibly, 2012; Tinkle, Wilbur, & Tilley, 1970). However, it is currently unknown whether the same also applies to oviparous and viviparous modes co‐occurring within the same species, and we were unable to quantify the age and past reproductive history of the mother lizards in this study.

Also in contrast with our hypothesis, the viviparous females had the longest telomeres, while the oviparous females had the shortest telomeres. In mammals, telomere length is generally phylogenetically conserved, although exceptions to this do exist (Gomes et al., 2011, 2010). It is unknown to what extent this applies to reptiles. One possibility is that differing telomere lengths in common lizards have evolved as an effect of the life‐history divergence among the reproductive modes. It is also possible that we wrongly presumed oviparity to carry a lesser maternal burden. We did not find a significant difference in maternal mass among the reproductive modes, and a study by Demarco and Guillette (1992) found viviparity to carry a minimal metabolic cost. Moreover, within a reproductive season, oviparous females might invest more in their offspring compared to viviparous females (Recknagel & Elmer, 2019). We found that ROM was larger for oviparous females compared to viviparous females, indicating that oviparous females provide their offspring with more nutrients (i.e. yolk). While we could not assess this here, the shorter time of pregnancy might also allow oviparous females to produce more than one clutch per year (Heulin, Guillaume, Vogrin, Surget‐Groba, & Tadic, 2000; Lindtke et al., 2010), resulting in an even larger reproductive effort per season for oviparous females.

It is worth noting that post‐embryonic telomere repair mechanisms, such as telomerase expression, have been documented in several lizard species to date (Alibardi, 2015; Ujvari et al., 2017). In addition, several studies on fish have found higher telomerase expression in actively dividing cells (Peterson, Mok, & Au, 2015; Yap, Yeoh, Brenner, & Venkatesh, 2005). We found that the viviparous offspring were significantly smaller at hatching but were of equivalent size at the maternal life stage, perhaps due to an increased rate of growth. Therefore, this possible divergence in telomere length between the reproductive modes may have occurred, in part because of differences in growth rate and its possible association with telomerase expression. Longer telomeres in viviparous common lizards might therefore have co‐evolved with (a) a smaller reproductive investment per season and (b) an increase in growth rate and potentially also longevity.

We do not know the age of the maternal lizards and can therefore not rule out possible age effects. It may be that viviparous common lizards differ in their longevity or their age at maturity, although there are currently no data to suggest this. Telomeres have been found to shorten in humans and other longer‐lived mammals and birds (Haussmann et al., 2003), in part because many larger, longer‐lived endotherm species appear to down‐regulate telomere repair mechanisms in post‐embryonic somatic tissues, perhaps as a tumour suppression mechanism (Gomes et al., 2010). However, the direction of the telomere–age relationship is less well established in ectothermic species, and studies have detected telomerase expression in a number of reptile, amphibian and fish species (Gomes et al., 2010; Simide, Angelier, Gaillard, & Stier, 2016; Ujvari et al., 2017). Therefore, we may have identified a difference in telomere length between the reproductive modes, in part because age at reproduction may also differ among the modes. However, age and body size are thought to be correlated in the common lizard (Richard, Lecomte, Fraipont, & Clobert, 2005) and we included body mass (highly correlated with body size) as a covariate in analyses. In addition, the exact same pattern was found at the offspring stage, when age was similar among the reproductive modes (i.e. days since copulation).

As with the adult females, we also found that the viviparous offspring had the longest telomeres, the oviparous offspring had the shortest telomeres, and the admixed offspring had intermediate telomere lengths. Had this embryonic development occurred in the wild, we might have hypothesized that the viviparous offspring had longer telomeres because viviparity is thought to confer more stable embryonic conditions (Vedder et al., 2018). However, all of the embryos in this study developed in stable incubator conditions. While there may still be some degree of variation in embryonic conditions (e.g. higher level of respiratory exchange in the viviparous mode), it is unlikely that this caused the observed differences in telomere length. The complexity of telomere heritability is still not fully understood in lizards. It could be that offspring inherit their telomere length via the initial telomere length of the fertilized egg (Dugdale & Richardson, 2018), presuming that viviparous mothers also have a longer germline telomere length. Additionally, offspring may also be inheriting the genetic information that controls telomere elongation during embryogenesis (Kalmbach, Robinson, Wang, Liu, & Keefe, 2014; Liu et al., 2007; Schaetzlein et al., 2004). For example, it is thought that these embryonic telomere elongation programmes may restore telomeres to a set length (Schaetzlein et al., 2004); therefore, it is possible that this genetic information has also diverged between the reproductive modes.

We still know relatively little about the determinants of species‐specific telomere length ranges, and how they may have evolved from species‐specific trade‐offs between long and short telomeres. It is often hard to disentangle such differences, in part because species differ significantly in the types of environment that they inhabit. However, by studying a unique common lizard population that exhibits both oviparous and viviparous reproduction in the same habitat, we have identified potential links between life‐history divergence and telomere length, suggesting that populations such as these may prove fruitful in future studies. It would also now be interesting to examine whether the difference in telomere length between oviparous and viviparous individuals may affect long‐term patterns of senescence and longevity.

## AUTHORS' CONTRIBUTIONS

D.M., H.R., K.R.E. and P.M. conceived of the idea for this study. H.R. led the field aspect of the study. D.M. and H.R. conducted laboratory analyses. D.M. and H.R. led data analysis and writing of the manuscript, with critical contribution from K.R.E. and P.M. All authors gave final approval for publication.

## Data Availability

Data are available via the Dryad Digital Repository https://doi.org/10.5061/dryad.712hc6m (McLennan, Recknagel, Elmer, & Monaghan, 2019).
