# Supplementary material for: Distinct telomere differences within a reproductively bimodal common lizard population
Source: Funct Ecol. 2019 Jul 30;33(10):1917–27. doi: 10.1111/1365-2435.13408 (PMC6853248; doi:10.1111/1365-2435.13408)
Supplement: Supplementary file 1 [file FEC-33-1917-s001.pdf]

Functional Ecology
BRITISH ECOLOGICAL SOCIETY

What can chromosome ends tell us about the cost of birthing live young?

Darryl McLennan, Hans Recknagel, Kathryn Elmer and Pat Monaghan

Within reptiles, giving birth to live young has evolved independently from egg laying about a hundred times. By retaining offspring within the womb for longer, live bearing mothers provide their young with a more stable and protected developmental environment. Less is known about the costs and benefits for the mother. However, it is presumed that live bearing may carry a greater cost for mothers, e.g. carrying offspring in the womb for longer periods may require more energy.

Comparing the effects of live bearing and egg laying on other traits is difficult, partly because most species have adopted only one of these strategies and comparing species is complicated due to evolutionary and ecological differences. However, by studying the European common lizard, one of the few vertebrates where both reproductive modes can be found within the same species, robust and controlled comparisons can be made.

In this study, we investigated whether telomere length differs between live bearing and egg laying common lizards. Telomeres cap chromosome ends and protect the central part of the chromosome that contains the active genes. Telomeres shorten during cell division; however, this shortening can be accelerated by exposure to conditions that generate stress within cells. Telomeres can shorten to such an extent that the genome becomes unstable. At this point, the cell ceases to function properly and often dies; therefore, telomere length is often considered a good indicator of biological state.

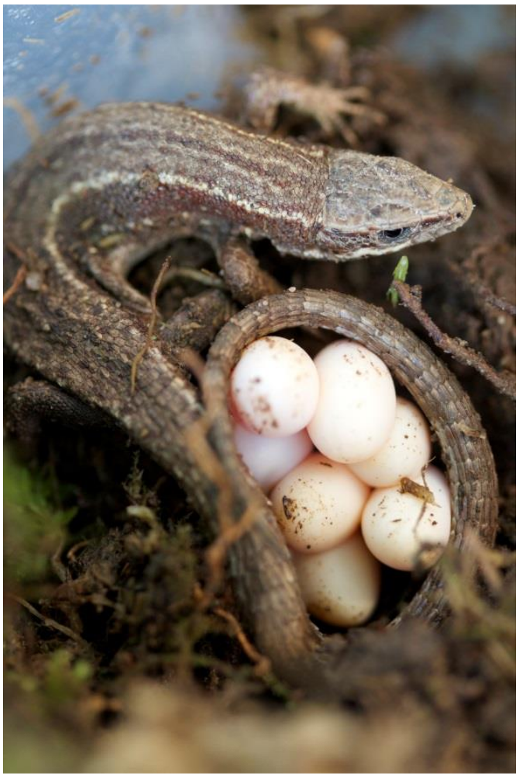

We initially hypothesised that live bearing mothers would have relatively shorter telomeres than egg laying mothers, since live bearing appears to require more effort. However, contrary to our predictions, both the live bearing mothers and their offspring had longer telomeres than egg laying mothers and offspring. That the difference is significant at the offspring stage suggests that it is not due to reproductive effort. It is possible that it is due to differences in the growth rates of the offspring. Alternatively, the two reproductive modes may have evolved differing telomere dynamics over time and so their 'starting' telomere length is different. How these telomere differences have coevolved with other life history traits remains to be investigated.
