# Supplementary material for: Distinct telomere differences within a reproductively bimodal common lizard population
Source: Funct Ecol. 2019 Jul 30;33(10):1917–27. doi: 10.1111/1365-2435.13408 (PMC6853248; doi:10.1111/1365-2435.13408)
Supplement: Supplementary file 2 [file FEC-33-1917-s002.docx]

**Supplementary Information corresponding to:**

**Distinct telomere differences within a reproductively bimodal common lizard population**

McLennan D. *^1,2^, Recknagel H. *^1^, Elmer K.R ^1^ and Monaghan P. ^1^

^1^ Institute of Biodiversity, Animal Health and Comparative Medicine, Graham Kerr Building, University of Glasgow, Glasgow, G12 8QQ, UK.

^2^ Department of Fish Ecology and Evolution, EAWAG, Seestrasse 79, 6047, Kastanienbaum, Switzerland

* These authors contributed equally to this work

Corresponding author: [darrylmclennan@outlook.com](mailto:darrylmclennan@outlook.com)

**SUPPLEMENTARY MATERIALS AND METHODS**

**ddRADSeq library preparation protocol**

We used a double-digest restriction site-associated sequencing (ddRADSeq) approach to genetically distinguish between the reproductive modes and admixed individuals. To do so, we followed the ddRADSeq library preparation protocol of Recknagel, Kamenos and Elmer (2018). All of the genomic libraries were sequenced at Edinburgh Genomics on an Illumina HiSeq 4000 machine with paired-end sequencing of 150 base pairs (bp). All of the generated reads were analyzed using STACKS software v.1.41 (Catchen *et al.* 2011) and trimmed to a common read length of 100 bp. The reads were then de-multiplexed and sorted into stacks of loci within each individual (maximum distance of 2 bp within a locus). Each individual was then aligned to a *Zootoca vivipara* reference genome v. 0.9 (Yurchenko, Recknagel & Elmer 2019) using Burrows-Wheeler transform (Li & Durbin 2010) and SAMtools (Li *et al.* 2009). A catalogue of all loci identified across individuals was subsequently created using the genome referenced stacks from each individual. Genotypes were extracted using the STACKS software, with a minimum coverage of 8x, presence in at least 50% of all individuals, and a minor allele frequency of 10%. The genomic ancestry of each female was then estimated by a structure clustering method, using ADMIXTURE v.1.3. (Alexander, Novembre & Lange 2009) with K=2.

**Telomere analysis PCR protocol**

Telomere PCR conditions were 15 min at 95°C followed by 27 cycles of 15 secs at 95°C, 30 secs at 58°C and 30 secs at 72°C. This was followed by a melt curve profile. RAG-1 PCR conditions were 15 min at 95°C followed by 40 cycles of 15 secs at 95°C, 30 secs at 60°C and 30 secs at 72°C. Again, this was followed by a melt curve profile. PCRs were performed on an Mx3005P qPCR system (Agilent).

The telomere (T) and single copy gene (S) assays were performed on separate 96 well plates, with each sample run in triplicate for each assay. In addition to the samples, each plate also included a six-fold serial dilution of a reference sample (1.25 - 40 ng/well), and a non-target control (NTC). The DNA for the serial dilution was a pool of DNA from 30 individuals and included both life stages (mother & offspring) and all reproductive modes (oviparous, viviparous and admixed). The NTCs contained all reaction components apart from DNA and were included on each plate (in triplicate) to check for non-specific binding and potential contamination between sample wells. Each reaction contained 12.5µl 2x ABsolute Blue qPCR SYBR Green Mix low ROX (Fisher Scientific), forward and reverse primers and DNA (wells containing sample, standard) or water (wells containing NTC) in a total volume of 25µl. Both assays were performed using 10ng of DNA. Primer concentrations were 500nM for the telomere assay (Tel1b and Tel2b) and 200nM for the RAG-1 assay (LizRAG1-F and LizRAG1-R).

The qPCR raw data was analysed using qBASE software for Windows (Hellemans *et al.* 2007). This software helps to control for differences in amplification efficiency between plates (assessed from the standard curve of each plate). In addition, three inter-run calibrators (three points from the standard curve) were included to help control for possible inter-run variation. We used the software to normalise each telomere relative quantity (RQ) by the RAG-1 RQ for that sample. Therefore, for each sample, the qBASE software produced a calibrated normalized relative quantity (CNRQ). This is similar to the T/S ratio described by (Cawthon 2002) but with greater control of the qPCR efficiency and inter-plate variation.

**References**

Alexander, D.H., Novembre, J. & Lange, K. (2009) Fast model-based estimation of ancestry in unrelated individuals. *Genome Research,* **19,** 1655-1664.

Catchen, J.M., Amores, A., Hohenlohe, P., Cresko, W. & Postlethwait, J.H. (2011) Stacks: Building and Genotyping Loci De Novo From Short-Read Sequences. *G3: Genes|Genomes|Genetics,* **1,** 171-182.

Cawthon, R.M. (2002) Telomere measurement by quantitative PCR. *Nucleic Acids Research,* **30,** e47.

Hellemans, J., Mortier, G., De Paepe, A., Speleman, F. & Vandesompele, J. (2007) qBase relative quantification framework and software for management and automated analysis of real-time quantitative PCR data. *Genome biology,* **8,** R19.

Li, H. & Durbin, R. (2010) Fast and accurate long-read alignment with Burrows–Wheeler transform. *Bioinformatics,* **26,** 589-595.

Li, H., Handsaker, B., Wysoker, A., Fennell, T., Ruan, J., Homer, N., Marth, G., Abecasis, G., Durbin, R. & Genome Project Data Processing, S. (2009) The Sequence Alignment/Map format and SAMtools. *Bioinformatics,* **25,** 2078-2079.

Recknagel, H., Kamenos, N.A. & Elmer, K.R. (2018) Common lizards break Dollo’s law of irreversibility: Genome-wide phylogenomics support a single origin of viviparity and re-evolution of oviparity. *Molecular Phylogenetics and Evolution,* **127,** 579-588.

Yurchenko, A.A., Recknagel, H. & Elmer, K.R. (2019) Chromosome-level assembly of the common lizard (*Zootoca vivipara*) genome. *bioRxiv***,** 520528.
